# Supplementary material for: CNEURO-201, an Anti-amyloidogenic Agent and σ1-Receptor Agonist, Improves Cognition in the 3xTg Mouse Model of Alzheimer’s Disease by Multiple Actions in the Pathology
Source: Int J Mol Sci. 2025 Feb 3;26(3):1301. doi: 10.3390/ijms26031301 (PMC11818425; doi:10.3390/ijms26031301)

SUPPLEMENTARY INFORMATION

CNEURO-201, an anti-amyloidogenic agent and σ1-receptor agonist improves cognition in the *3xTg* mouse model of Alzheimer's disease by multiple actions in the pathology

Martínez-Orozco Humberto [1], Bencomo-Martínez Alberto [2], Maya-Arteaga Juan Pablo [1], Rubio-De Anda Pedro Francisco [1], Sanabria-Romero Fausto [1], Mena-Casas Zyanya Gloria [1], Rodríguez-Vargas Isaac [1], Hernández-Puga Ana Gabriela [3], Menéndez-Soto del Valle Roberto [2], Sablón-Carrazana Marquiza [2], Rodríguez-Tanty Chryslaine [2], and Díaz-Cintra Sofía [1, *]

[1] Departamento de Neurobiología del Desarrollo y Neurofisiología, Instituto de Neurobiología-UNAM Campus Juriquilla, Boulevard Juriquilla 3001, C.P 76230, Querétaro, México
[2] Departamento de Farmacología, Centro de Neurociencias de Cuba, Avenida Independencia 8126, La Habana, Cuba
[3] Centro de Investigación Biomédica Avanzada, Facultad de Medicina, Universidad Autónoma de Querétaro, Carretera a Chichimequillas S/N, C.P. 76140, Querétaro, México
* Author to whom correspondence should be addressed: yoldi@unam.mx

SUPPLEMENTARY INFORMATION

Table of Contents

	page
**1.- *In silico* studies. Molecular dynamics simulations.**	
**Figure S1.** Temporal stabilities of donepezil-AChE complexe (rigth) and CNEURO-201-AChE (left) by means of spatial positions (top) (Root Mean Square Deviation-RMSD), and energies (Coulomb, red; Lennard-Jones-LJ, blue; bottom) obtained in the MD studies.	3
**Figure S2.** Total contacts expressed in percent between AChE enzyme amino acids and donepezil (top) and CNEURO-201 (bottom) at 5 Å, during 200 ns of simulation.	4
**Figure S3.** Total hydrogens bonds between AChE enzyme amino acids and donepezil (top) and CNEURO-201 (bottom) at 5 Å, during 200 ns of simulation.	5

In silico studies.

Figure S1. Temporal stabilities of donepezil-AChE complexe (rigth) and CNEURO-201-AChE (left) by means of spatial positions (top) (Root Mean Square Deviation-RMSD), and energies (Coulomb, red; Lennard-Jones-LJ, blue; bottom) obtained in the MD studies.

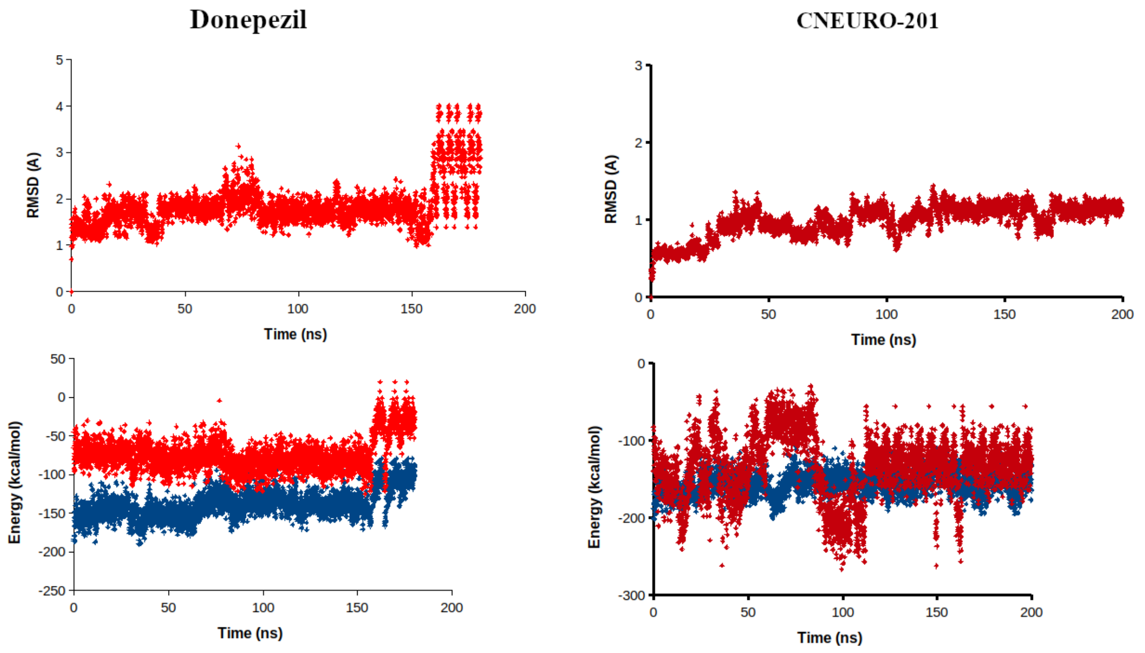

Figure. S2. Total contacts expressed in percent between AChE enzyme amino acids and donepezil (top) and CNEURO-201 (bottom) at 5 Å, during 200 ns of simulation.

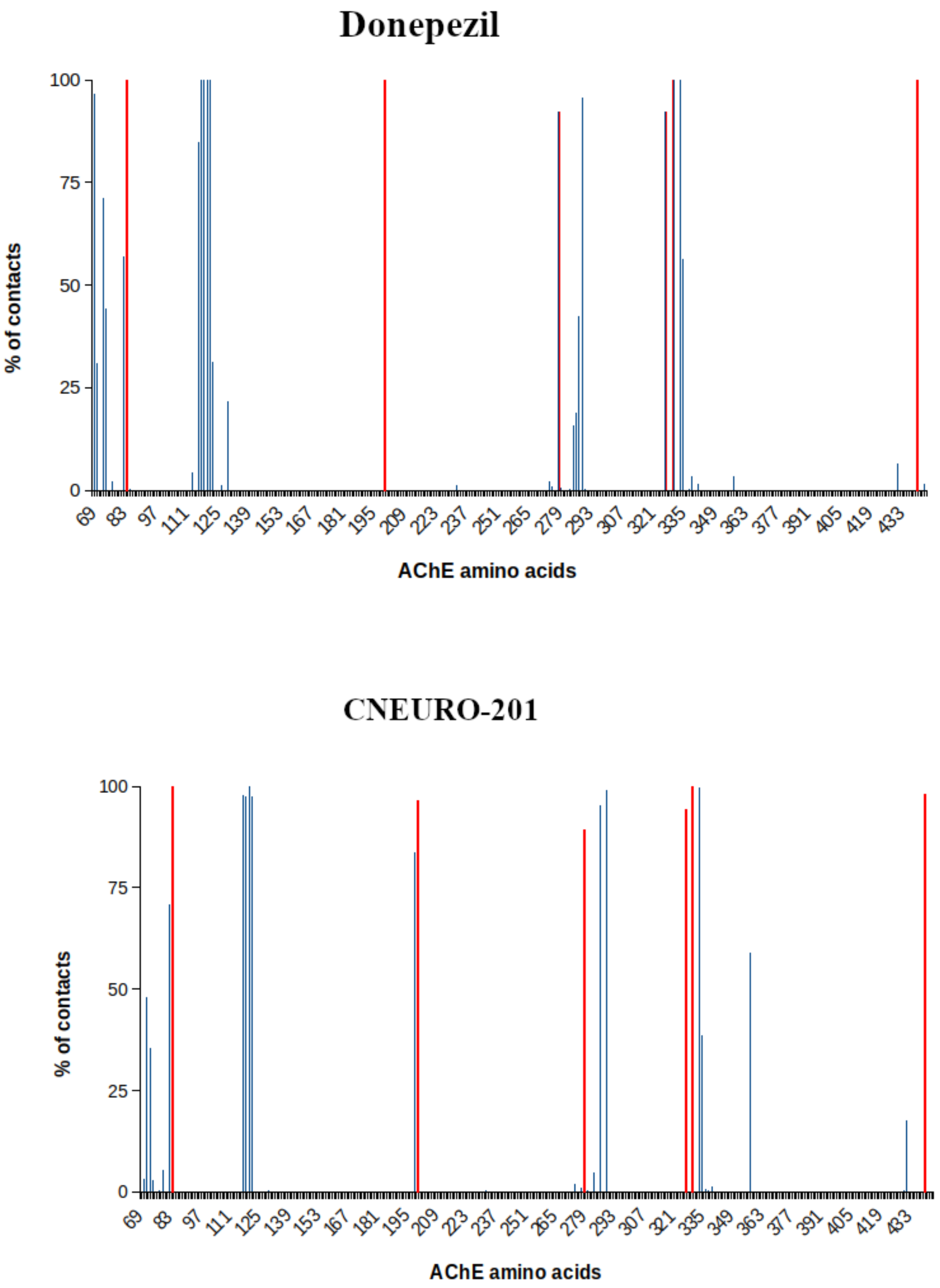

Figure S3. Total hydrogens bonds between AChE enzyme amino acids and donepezil (top) and CNEURO-201 (bottom) at 5 Å, during 200 ns of simulation.

Donepezil

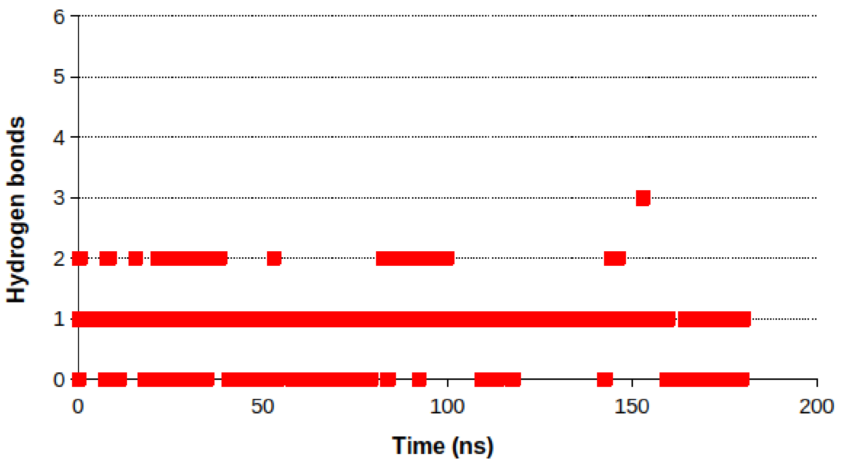

CNEURO-201

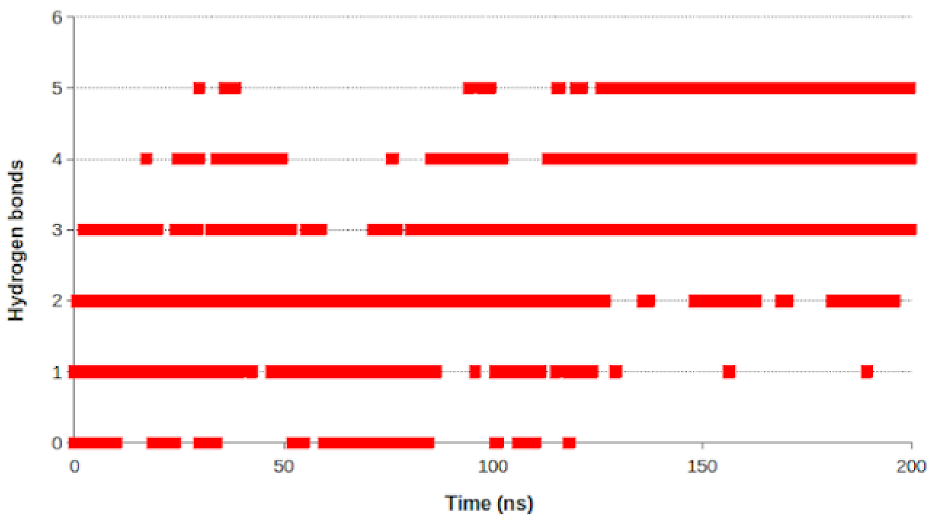

Supplement: Supplementary file 1 [file ijms-26-01301-s001.zip › ijms-3401897-supplementary.pdf]
